# Association between vitamin D receptor polymorphisms and acute pancreatitis: A protocol for systematic review and meta analysis

**DOI:** 10.1097/MD.0000000000025508

**Published:** 2021-04-23

**Authors:** Xiaofeng Li, Xianghai Gan, Junzuo Gong, Tianyi Mou, Hua Zhou, Mengqin Li

**Affiliations:** a Department of Emergency; b Dental Department, Affiliated Hospital of North Sichuan Medical College, Nanchong, Sichuan Province, China.

**Keywords:** acute pancreatitis, polymorphism, protocol, meta-analysis, vitamin D receptor

## Abstract

**Background:**

Several studies have been performed to investigate the association between *vitamin D receptor* (*VDR*) gene polymorphism and acute pancreatitis, but the results are inconclusive. We conducted this meta-analysis for a precise estimation of the association between BsmI (rs1544410), ApaI (rs7975232), TaqI (rs731236), and FokI (rs2228570) polymorphisms and acute pancreatitis.

**Methods:**

Appropriate studies were retrieved by searching Web of Science, PubMed, Scopus, and Google scholar databases, until January 31, 2021. Two reviewers independently conducted data extraction and literature quality evaluation. Odds ratios and 95% confidence intervals were calculated to evaluate the strength of the association.

All of the data were analyzed with Stata 16.0.

**Results:**

The results of this meta-analysis will be submitted to a peer-reviewed journal for publication.

**Conclusions:**

This meta-analysis will summarize the association between BsmI, ApaI, TaqI, and FokI polymorphisms and the risk of acute pancreatitis.

**Ethics and dissemination:**

Ethical approval was not required for this study. The systematic review will be published in a peer-reviewed journal, presented at conferences, and shared on social media platforms.

**OSF REGISTRATION NUMBER:**

DOI 10.17605/OSF.IO/83W7R.

## 1 Introduction

Acute pancreatitis (AP) is a common acute abdomen characterized by acute abdominal pain, abdominal distension, nausea, vomiting, abdominal tenderness, and rebound pain.^[1,2]^ A large number of studies have revealed that AP is related to a variety of factors, including excessive drinking, high blood lipids, metabolic disorders, pancreatic duct obstruction, virus or bacterial infection, and so on.^[3–7]^ However, not all individuals who are exposed to similar risk factors will develop AP, indicating that genetic factors play an important role in the occurrence and development of AP.

Vitamin D (VD) dysregulation was proposed to be associated with the production of AP.^[8]^ The imbalance of active VD level is a major cause of hypercalcemia that is a risk element for AP. Due to polymorphisms in the *VDR* gene, the impairment of *VDR* can lead to a significant effect on the equilibrium of VD levels in the circulation and on its activity in the body. Moreover, Serum 25-hydroxyvitamin D3(25-(OH) D3) level was inversely related to the severity of AP and inflammatory markers such as C-reactive protein. VD also affects the innate and adaptive immune responses and reduces oxidative stress, which could initiate AP.^[9]^

In recent years, there are more and more studies on the relationship between *VDR* polymorphism and the risk of AP, but the results of these studies are not consistent.^[10,11]^ In this study, we performed a systematic review and a meta-analysis to evaluate the association between different genetic variants of *VDR* gene and AP, and to evaluate the association of BsmI, ApaI, TaqI, and FokI polymorphisms within different ethnic subgroups. The results were pooled together to obtain reliable conclusions.

## 2 Methods

### 2.1 Study registration

The protocol of this review was registered in OSF (OSF registration number: DOI 10.17605/OSF.IO/83W7R). It was reported to follow the statement guidelines of preferred reporting items for systematic reviews and meta-analyses protocol.^[12]^

### 2.2 Inclusion criteria

Qualified studies have to meet the following criteria:

(1) Predefined diagnosis criteria for AP;
(2) Case-control studies evaluated the association between BsmI, ApaI, TaqI, and FokI polymorphism and AP risk;
(3) Provided detailed genotype frequencies;
(4) If multiple reports based on the same study population were available, the most recent or largest study will be selected.

### 2.3 Exclusion criteria

The main exclusion criteria are as follows:

(1) Insufficient data offered to calculate Odds ratio (OR) estimate;
(2) Non-case-control studies;
(3) Duplicate data from a same cohort.

### 2.4 Search strategy

Two investigators independently performed a systematically computerized search for English studies through Web of Science, PubMed, Scopus, and Google Scholar databases up to January 31, 2021. The keywords for searching included the combination of “*vitamin D receptor*, *VDR*, polymorphism, BsmI, rs1544410, ApaI, rs7975232, TaqI, rs731236, FokI, rs2228570, pancreatitis, acute pancreatitis.” Furthermore, studies were identified through manual searches on reviews and retrieved studies. The search strategy for PubMed is displayed in Table 1, and the corresponding keywords would be applied in other databases.

**Table 1:** Search strategy in PubMed database.

Number	Search terms
#1	Pancreatitis[MeSH]
#2	Pancreatitides[Title/Abstract]
#3	or/1–2
#4	Receptors, Calcitriol[MeSH]
#5	Calcitriol Receptors[Title/Abstract]
#6	Cholecalciferol Receptors[Title/Abstract]
#7	Receptors, Vitamin D[Title/Abstract]
#8	Vitamin D 3 Receptors[Title/Abstract]
#9	Vitamin D Receptors[Title/Abstract]
#10	1,25-Dihydroxycholecalciferol Receptor[Title/Abstract]
#11	1,25-Dihydroxycholecalciferol Receptors[Title/Abstract]
#12	1,25-Dihydroxyvitamin D 3 Receptor[Title/Abstract]
#13	1,25-Dihydroxyvitamin D3 Receptor[Title/Abstract]
#14	1,25-Dihydroxyvitamin D3 Receptors[Title/Abstract]
#15	Calcitriol Receptor[Title/Abstract]
#16	Receptors, 1,25-Dihydroxyvitamin D 3[Title/Abstract]
#17	Receptors, Cholecalciferol[Title/Abstract]
#18	Receptors, Vitamin D 3[Title/Abstract]
#19	Receptors, Vitamin D3[Title/Abstract]
#20	Vitamin D 3 Receptor[Title/Abstract]
#21	Vitamin D Receptor[Title/Abstract]
#22	Vitamin D3 Receptor[Title/Abstract]
#23	Vitamin D3 Receptors[Title/Abstract]
#24	1,25 Dihydroxycholecalciferol Receptor[Title/Abstract]
#25	1,25 Dihydroxycholecalciferol Receptors[Title/Abstract]
#26	1,25 Dihydroxyvitamin D 3 Receptor[Title/Abstract]
#27	1,25 Dihydroxyvitamin D3 Receptor[Title/Abstract]
#28	1,25 Dihydroxyvitamin D3 Receptors[Title/Abstract]
#29	D Receptor, Vitamin[Title/Abstract]
#30	D Receptors, Vitamin[Title/Abstract]
#31	D3 Receptor, 1,25-Dihydroxyvitamin[Title/Abstract]
#32	D3 Receptor, Vitamin[Title/Abstract]
#33	D3 Receptors, 1,25-Dihydroxyvitamin[Title/Abstract]
#34	D3 Receptors, Vitamin[Title/Abstract]
#35	Receptor, 1,25-Dihydroxycholecalciferol[Title/Abstract]
#36	Receptor, 1,25-Dihydroxyvitamin D3[Title/Abstract]
#37	Receptor, Calcitriol[Title/Abstract]
#38	Receptor, Vitamin D[Title/Abstract]
#39	Receptor, Vitamin D3[Title/Abstract]
#40	Receptors, 1,25-Dihydroxycholecalciferol[Title/Abstract]
#41	Receptors, 1,25-Dihydroxyvitamin D3[Title/Abstract]
#42	or/4–41
#43	polymorph∗[Title/Abstract]
#44	susceptibility[Title/Abstract]
#45	or/43–44
#46	#3 and #42 and #45

### 2.5 Data collection and analysis

#### 2.5.1 Selection of studies

**Figure 1:**
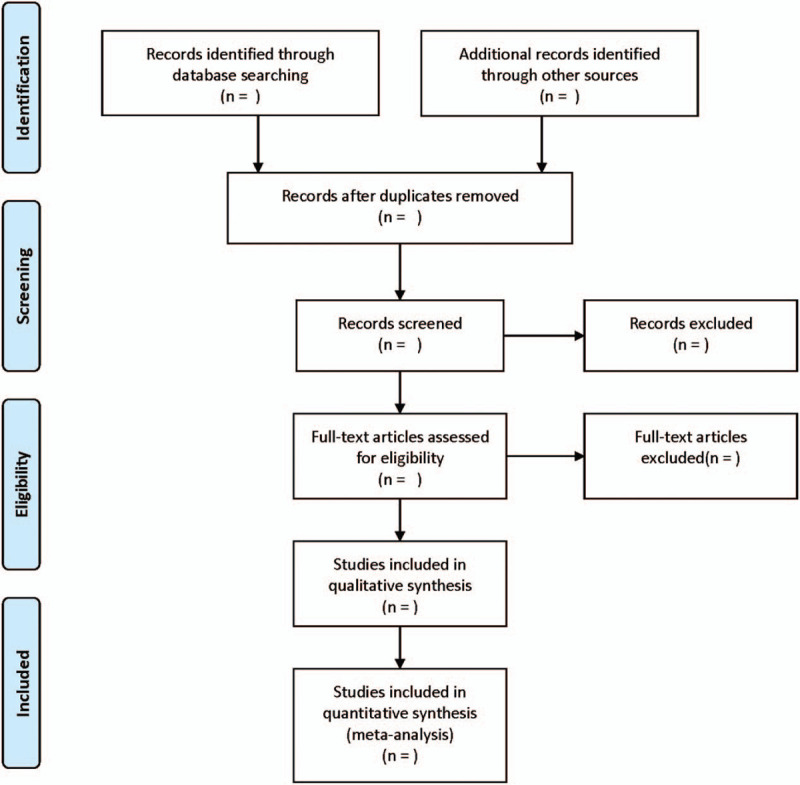
Flow diagram of study selection process.

Two reviewers complete the screening process independently, and any differences are decided by a third reviewer. The screening process of the article includes reading the title, the abstract, and the full text, so as to determine whether it meets the inclusion criteria. The researchers record the reasons to exclude each study in light of the preferred reporting items for systematic reviews and meta-analysis guidelines and report the screening results as well. The flowchart is exhibited in Figure 1.

#### 2.5.2 Data extraction

The collected information from each article are as follows: The first author and year, country, region, race, gene frequency of case group and control group, source of control group, *P* value for Hardy–Weinberg equilibrium (HWE) of controls, single nucleotide polymorphism detection method and so on. If the extracted data are inconsistent, discussion should be made to solve it.

#### 2.5.3 Methodology quality assessment

Two authors independently assessed the quality of the included studies based on Newcastle Ottawa Scale.^[13]^ This scale uses a star rating system to judge the methodological quality and consists of three parts, namely selection, comparability, and ascertainment of exposure. The full score is 9 stars, and the score of 5 or more is regarded as “high quality.” Otherwise, the study is regarded as “low quality.”^[14]^ Any disagreements on the Newcastle-Ottawa scale score of the studies were resolved through a comprehensive reassessment by other authors.

#### 2.5.4 Dealing with missing data

The reason for the loss of data in the period of data screening and extraction is identified here. If the data of potential studies are insufficient, missing, or vague, we would attempt to contact the authors. These studies would be excluded only if the data are not available through the method described above.

#### 2.5.5 Statistical analysis

Crude Odds ratio (ORs) with corresponding 95% confidence intervals were used to estimate the strength of the association between the BsmI, ApaI, TaqI, and FokI polymorphism and AP risk. The statistical significance of the pooled ORs was examined by performing *Z* test and *P* < .05 was considered as statistically significant. In the meta-analysis, the overall pooled ORs were calculated for four models, including the allelic, dominant, recessive, and homozygous models. Deviation from HWE was examined by carrying out Chi-square test and *P* value < .05 indicated a departure from HWE. The between-study heterogeneity was assessed by the *I*^2^ statistic, which was calculated from the Q statistic. If the heterogeneity was statistically significant (*P* < .05 for *Q*-test or I^2^ > 50%), the random-effects model (based on DerSimonian-Laird method) was adopted to get the pooled estimates. Otherwise, the fixed-effects model (based on Mantel–Haenszel method) was adopted. All of the statistical analyses were conducted by STATA 16.0 (StataCorp, College Station, TX), and the *P* values were 2-sided.

#### 2.5.6 Subgroup analysis

We will conduct subgroup analyses based on age, ethnicity, and disease severity

#### 2.5.7 Sensitivity analysis

The eligible study was sequentially removed to perform the sensitivity analysis.

#### 2.5.8 Assessment of publication biases

Publication bias was assessed by Begg rank correlation and Egger linear regression. The publication bias was regarded as statistically difference when *P* < .05.^[15,16]^

#### 2.5.9 Ethics and dissemination

The content of this article does not involve moral approval or ethical review and would be presented in print or at relevant conferences.

## 3 Discussion

In recent years, studies on the relationship between inflammatory mediators and the risk of AP have proved that the severe systemic inflammatory response triggered by AP is not mediated by a single vector, but has complex pathophysiological pathways. Among these pathways, oxidative stress and cytokines are particularly important.^[17]^

The 25-(OH) D3 participates in human immune and inflammatory response.^[18]^ Kim et al discovered that the concentration of 25-(OH) D3 in AP dogs was significantly lower than that in healthy dogs.^[19]^ Active VD metabolite, and 1,25-dihydroxyvitamin D selectively bind to specific *VDR*.^[20]^ Afterward, the *VDR* regulates the transcription of genes that are involved in calcium metabolism, cell proliferation and differentiation, senescence, and *t*-cell-mediated immune response.^[21–28]^*VDR* is also expressed in pancreatic β cells and may play an important role in maintaining normal insulin levels consistent with glucose concentration and glucose tolerance.^[29–31]^ The polymorphism of *VDR* may have key effects on the balance of circulating VD concentrations and the activity of final metabolites throughout the body, because VD works through *VDR*.

So far, although many researchers have focused on the relationship between *VDR* polymorphism and AP susceptibility, the cumulative evidence of this association has not been systematically evaluated. In this study, we will conduct a systematic review and meta-analysis to combine the results of numerous studies and generate more reliable risk association estimation to guide the prevention and treatment of AP. The advantages of this study are as follows:

(1) Large data sets from all eligible latest studies will be merged;
(2) In order to explore heterogeneity, we will try to avoid post-subgroup analysis;
(3) Sensitivity analysis will be conducted on each genetic model for the improvement of the reliability of the results. Therefore, the release protocol will avoid potential biases related to data mining as much as possible and help to obtain convincing evidence.

## Author contributions

**Conceptualization:** Mengqin Li.

**Data curation:** Xiaofeng Li, Xianghai Gan.

**Formal analysis:** Mengqin Li, Xianghai Gan, Junzuo Gong.

**Methodology:** Xianghai Gan, Tianyi Mou.

**Project administration:** Mengqin Li.

**Resources:** Junzuo Gong.

**Software:** Junzuo Gong, Hua Zhou.

**Supervision:** Mengqin Li.

**Validation:** Tianyi Mou, Hua Zhou, Xiaofeng Li.

**Visualization:** Xiaofeng Li, Hua Zhou.

**Writing – original draft:** Mengqin Li, Xiaofeng Li.

**Writing – review & editing:** Mengqin Li, Xiaofeng Li.

## References

[1] Talley N. Practice Parameters Committee of the American College of Gastroenterology Guidelines for the management of dyspepsia. Am J Gastroenterol 2005;101: 2379-2000. 10.1111/j.1572-0241.2005.00225.x 16181387

[2] Pandol SJ Saluja AK Imrie CW. Acute pancreatitis: bench to the bedside. Gastroenterology 2007;132:1127–51. 17383433 10.1053/j.gastro.2007.01.055

[3] Álvarez J Castro P Fernández M. Clinical and radiological indicators of severity in patients with acute pancreatitis. Boletín De La Asociación Médica De Puerto Rico 2015;107:33–7. 26035982

[4] Greer JB Thrower E Yadav D. Epidemiologic and mechanistic associations between smoking and pancreatitis. Curr Treat Options Gastroenterol 2015;13:332–46. 26109145 10.1007/s11938-015-0056-9 PMC4532333

[5] Tun-Abraham ME Obregón-Guerrero G Romero-Espinoza L. Acute pancreatitis associated with hypercalcaemia. Cirugia Y Cirujanos 2015;83:227–31. 26123156 10.1016/j.circir.2015.05.006

[6] Greep JM Wielinga WJ Rijnders WP. Acute pancreatitis. Nederlands Tijdschrift Voor Geneeskunde 1960;104:2641–5. 13708375

[7] Pohl JF Uc A. Paediatric pancreatitis. Curr Opin Gastroenterol 2015;31:380–6. 26181572 10.1097/MOG.0000000000000197 PMC4732740

[8] Li J Zhou R Zhang J. Calcium signaling of pancreatic acinar cells in the pathogenesis of pancreatitis. World J Gastroenterol 2014;20:16146–52. 25473167 10.3748/wjg.v20.i43.16146 PMC4239501

[9] Huh JH Kim JW Lee KJ. Vitamin D deficiency predicts severe acute pancreatitis. United Eur Gastroenterol J 2019;7:90–5. 10.1177/2050640618811489 PMC6374843 30788120

[10] El-Mahdy RI Ramadan HK Mohammed H. Impact of the etiology and Vitamin D receptor TaqI rs731236 gene polymorphism on the severity of acute pancreatitis. J Hepato-biliary-pancreat Sci 2020;27:896–906. 10.1002/jhbp.817 32780933

[11] Cieślińska A Kostyra E Fiedorowicz E. Single nucleotide polymorphisms in the vitamin D receptor gene (VDR) may have an impact on acute pancreatitis (AP) development: a prospective study in populations of AP patients and alcohol-abuse controls. Int J Mol Sci 2018;19:1919. 10.3390/ijms19071919 PMC6073954 29966312

[12] Shamseer L Moher D Clarke M. Preferred reporting items for systematic review and meta-analysis protocols (PRISMA-P) 2015: elaboration and explanation. BMJ (Clinical research ed) 2015;350:g7647. 10.1136/bmj.g7647 25555855

[13] Stang A. Critical evaluation of the Newcastle-Ottawa scale for the assessment of the quality of nonrandomized studies in meta-analyses. Eur J Epidemiol 2010;25:603–5. 20652370 10.1007/s10654-010-9491-z

[14] Zhang Q Jin Y Li X. Plasminogen activator inhibitor-1 (PAI-1) 4G/5G promoter polymorphisms and risk of venous thromboembolism - a meta-analysis and systematic review. VASA Zeitschrift fur Gefasskrankheiten 2020;49:141–6. 31920171 10.1024/0301-1526/a000839

[15] Lewis SJ Zammit S Gunnell D. Bias in meta-analysis detected by a simple, graphical test. BMJ Clin Res 1997;315:629–34. 10.1136/bmj.315.7109.629 PMC2127453 9310563

[16] Duval S Tweedie R. Trim and fill: a simple funnel-plot-based method of testing and adjusting for publication bias in meta-analysis. Biometrics 2000;56:455–63. 10877304 10.1111/j.0006-341x.2000.00455.x

[17] Zhang XP Li ZJ Zhang J. Inflammatory mediators and microcirculatory disturbance in acute pancreatitis. Hepatobiliary Pancreat Dis Int 2009;8:351–7. 19666402

[18] Schauber J Dorschner RA Coda AB. Injury enhances TLR2 function and antimicrobial peptide expression through a vitamin D–dependent mechanism. J Clin Investig 2007;117:803–11. 17290304 10.1172/JCI30142 PMC1784003

[19] Kim DI Kim H Son P. Serum 25-hydroxyvitamin D concentrations in dogs with suspected acute pancreatitis. J Vet Med Sci 2017;79:1366–73. 28659537 10.1292/jvms.16-0647 PMC5573823

[20] Holick MF. Vitamin D deficiency. N Engl J Med 2007;357:266–81. 17634462 10.1056/NEJMra070553

[21] Haussler MR Whitfield GK Haussler CA. The nuclear vitamin D receptor: biological and molecular regulatory properties revealed. J Bone Miner Res 1998;13:325–49. 9525333 10.1359/jbmr.1998.13.3.325

[22] Mehta RG Mehta RR. Vitamin D and cancer. J Nutr Biochem 2002;13:252–64. 12015155 10.1016/s0955-2863(02)00183-3

[23] Mathieu C van Etten E Decallonne B. Vitamin D and 1,25-dihydroxyvitamin D3 as modulators in the immune system. J Steroid Biochem Mol Biol 2004;89–90:449–52. 10.1016/j.jsbmb.2004.03.014 15225818

[24] Valdivielso JM Fernandez E. Vitamin D receptor polymorphisms and diseases. Clin Chim Acta 2006;371:01–12. 10.1016/j.cca.2006.02.016 16563362

[25] Sayeed I Stein DG. Progesterone as a neuroprotective factor in traumatic and ischemic brain injury. Prog Brain Res 2009;175:219–37. 19660659 10.1016/S0079-6123(09)17515-5

[26] Ramagopalan SV Heger A Berlanga AJ. A ChIP-seq defined genome-wide map of vitamin D receptor binding: associations with disease and evolution. Genome Res 2010;20:1352–60. 20736230 10.1101/gr.107920.110 PMC2945184

[27] Sigmundsdottir H. From the bench to the clinic: new aspects on immunoregulation by vitamin D analogs. Dermato-endocrinology 2011;3:187–92. 22110778 10.4161/derm.3.3.15115 PMC3219169

[28] Harms LR Burne TH Eyles DW. Vitamin D and the brain. Best Pract Res Clin Endocrinol Metab 2011;25:657–69. 21872806 10.1016/j.beem.2011.05.009

[29] Johnson JD Kuang S Misler S. Ryanodine receptors in human pancreatic beta cells: localization and effects on insulin secretion. FASEB journal: official publication of the Federation of American Societies for Experimental Biology 2004;18:878–80. 15033925 10.1096/fj.03-1280fje

[30] Ishida H Norman AW. Demonstration of a high affinity receptor for 1,25-dihydroxyvitamin D3 in rat pancreas. Mol Cell Endocrinol 1988;60:109–17. 2850952 10.1016/0303-7207(88)90169-4

[31] Johnson JA Grande JP Roche PC. Immunohistochemical localization of the 1,25(OH)2D3 receptor and calbindin D28k in human and rat pancreas. Am J Physiol 1994;267 (3 Pt 1):E356–60. 7943215 10.1152/ajpendo.1994.267.3.E356

